# Needle in a haystack: an unusual case of video capsule endoscope retention

**DOI:** 10.1055/a-2094-9124

**Published:** 2023-06-12

**Authors:** Jiayinaer Bolatihan, Lingxi Lin, Yujen Tseng, Pinghong Zhou, Lili Ma

**Affiliations:** 1 Endoscopy Center and Endoscopy Research Institute, Zhongshan Hospital, Fudan University, Shanghai, China; 2 Department of Digestive Diseases, Huashan Hospital, Fudan University, Shanghai, China; 3 Endoscopy Center, Wusong Branch of Zhongshan Hospital, Fudan University, Shanghai, China

A patient was admitted with a 1-month history of intermittent abdominal pain, often associated with pre-prandial exacerbation. Then, 2 days prior to admission, the patient experienced hematochezia, with a rapid decline in hemoglobin level from 131 g/L to 80 g/L to 61 g/L. The patient underwent gastroscopy and colonoscopy, but no obvious cause of gastrointestinal bleeding was found.

Considering the relative short course of disease, with no symptomatic evidence of obstruction, video capsule endoscopy (VCE) was ordered to determine the cause of obscure gastrointestinal bleeding. The VCE was completed in 11:14 hours but did not complete the entire small-bowel examination and failed to pass after 72 hours. A computed tomography scan revealed that the VCE device was retained in the small bowel, located in the left upper quadrant of the abdomen ( Fig. 1 a, b ).

**Fig. 1:**
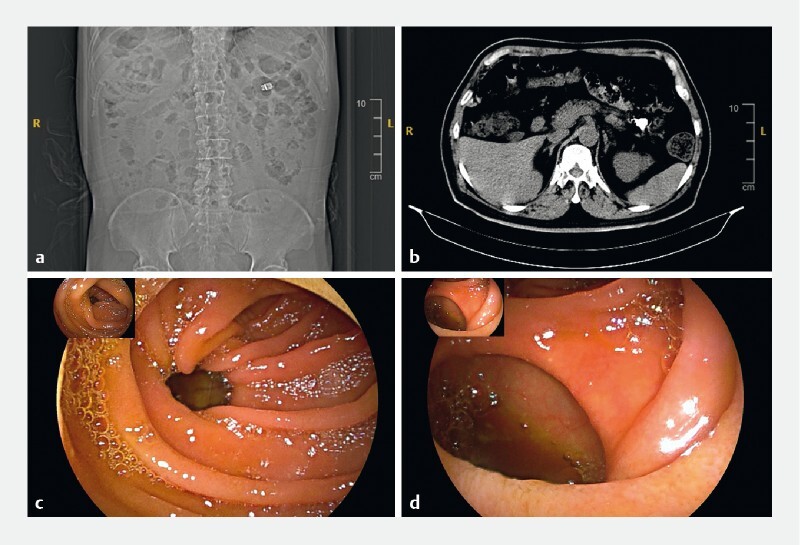
Investigations to locate the video capsule endoscope. **a, b** Radiology studies showed retention of the device in the small bowel. **c, d** Antegrade double-balloon enteroscopy revealed multiple diverticula in the jejunum.

A double-balloon enteroscopy (DBE) was performed to retrieve the VCE device. An antegrade approach revealed multiple diverticula in the jejunum ( Fig. 1 c, d ). Initially, the location of the VCE device was difficult to identify, but it was later found within a large diverticulum located in the upper segment of the jejunum. Unlike extraction of video capsules in stricturing or obstructive diseases, extraction of a foreign object from a diverticulum is technically difficult due to sharp angulation of the scope and accurate snare positioning. After multiple attempts and repeated adjustment, the VCE device was finally retrieved ( Video 1 ).

**Video 1** Retention of a video capsule endoscope in a jejunal diverticulum, retrieved with a polypectomy snare.


As VCE is noninvasive and convenient, it has become a common diagnostic tool for the investigation of obscure gastrointestinal bleeding and other small-bowel disorders 1 . However, VCE device retention is a known adverse event, with a reported incidence of approximately 2 %–10 %. Although rare, device retention may cause severe life-threatening complications. The majority of cases of video capsule retention are due to stricturing diseases, such as Crohn’s disease, or obstructing disease, such as small-bowel tumors 2 . VCE device retention in diverticular disease is thus an even rarer complication. Device retention may not require immediate intervention, but prolonged retention may require surgical extraction. This report described a case of VCE device retention in a jejunal diverticulum, which was successfully retrieved with DBE.

Endoscopy_UCTN_Code_TTT_1AP_2AB

## Notes

Endoscopy E-Videos

https://eref.thieme.de/e-videos
*E-Videos* is an open access online section of the journal *Endoscopy* , reporting on interesting cases and new techniques in gastroenterological endoscopy. All papers include a high-quality video and are published with a Creative Commons CC-BY license. Endoscopy E-Videos qualify for HINARI discounts and waivers and eligibility is automatically checked during the submission process. We grant 100% waivers to articles whose corresponding authors are based in Group A countries and 50% waivers to those who are based in Group B countries as classified by Research4Life (see: https://www.research4life.org/access/eligibility/ ). This section has its own submission website at https://mc.manuscriptcentral.com/e-videos
